# Toxicity profile of approved anti-PD-1 monoclonal antibodies in solid tumors: a systematic review and meta-analysis of randomized clinical trials

**DOI:** 10.18632/oncotarget.13315

**Published:** 2016-11-11

**Authors:** Ricardo Costa, Benedito A. Carneiro, Mark Agulnik, Alfred W. Rademaker, Sachin G. Pai, Victoria M. Villaflor, Massimo Cristofanilli, Jeffrey A. Sosman, Francis J Giles

**Affiliations:** ^1^ Division of Hematology/Oncology, Northwestern University Feinberg School of Medicine, Chicago, Illinois, USA; ^2^ Robert H. Lurie Comprehensive Cancer Center of Northwestern University, Chicago, Illinois, USA; ^3^ Northwestern University Department of Preventive Medicine, Chicago, Illinois, USA

**Keywords:** anti-PD1 antibodies, adverse events, meta-analysis, hypothyroidism, pruritus

## Abstract

**Purpose:**

Nivolumab and pembrolizumab are antibodies against the programmed-death-receptor- 1 (PD-1) which are associated with distinct immune related adverse effects (AEs). This meta-analysis of randomized clinical trials aims to summarize current knowledge regarding the toxicity profile of these agents.

**Methods:**

PubMed search was conducted in February of 2016. The randomized trials needed to have at least one of the study arms consisting of nivolumab or pembrolizumab monotherapy and a control arm containing no anti-PD-1 therapy. Data were analyzed using random effects meta-analysis for risk ratios. Heterogeneity across studies was analyzed using Q and I^2^ statistics.

**Results:**

Nine randomized trials and 5,353 patients were included in our meta-analysis. There was evidence of significant heterogeneity between studies. The pooled relative risk (RR) for treatment-related all grade AEs and grade 3/4 AEs was 0.88 (95% CI 0.81-0.95;*P*=0.002) and 0.39 (95% CI 0.29-0.53; *P*<0.001) respectively favoring anti-PD-1 therapy versus standard of care approach. The RR of treatment-related death was 0.45 (95% CI 0.19-1.09; *P*=0.076). Patients treated with PD-1 inhibitors had an increased risk of hyperthyroidism [RR of 3.44 (95% CI 1.98-5.99; *P*<0.001)] and hypothyroidism [RR of 6.79 (95% CI 3.10-14.84; *P*<0.001)]. All grade pruritus and vitiligo were also more common among these patients. The pooled absolute risks of pneumonitis and hypophysitis were 2.65% and 0.47% respectively.

**Conclusion:**

Approved PD-1 inhibitors are well tolerated, associated with significant low risk of severe treatment-related AEs and increased risk of thyroid dysfunction, pruritus, and vitiligo.

## INTRODUCTION

In recent years, antibodies against the programmed-death-receptor- 1 (PD-1) have shown remarkable therapeutic success for a subset of patients with advanced malignancies including melanoma, non-small cell lung cancer (NSCLC) and renal cell carcinoma (RCC) leading to their FDA approval. The efficacy of these drugs relies on enhancing anti-tumor immunity through the inhibition of negative regulatory signaling in T cells. In 2015, numerous clinical trials were reported demonstrating the efficacy of anti-PD-1 antibodies and leading to FDA approval of nivolumab for patients with advanced melanoma, RCC, and NSCLC alongside pembrolizumab for treatment of advanced melanoma and NSCLC. [1–5] The safety and efficacy of these compounds were compared to a variety of standard care therapies and by nature of their mechanism of action they are associated with a distinct toxicity profiles different from chemotherapy or targeted therapy.

The inhibition of immune checkpoint receptors can disrupt immune tolerance resulting in enhanced immune activation in normal tissue sites with significant toxicity. [6] The basis for the majority of these adverse events is an unregulated activation of T-cells directed at normal tissues. [7] T cell lymphocytic infiltrates are commonly observed in the setting of anti-PD-1 antibody associated pneumonitis. [8, 9] Many oncologists are not familiar with the clinical management of immune-related adverse events (irAEs). irAEs to anti-PD-1 monoclonal antibodies affects a wide range of organs including endocrine organs, thyroid, adrenal gland and pituitary, skin with rash or vitiligo, gastrointestinal tract, lung, kidney, liver, pancreas and the nervous system. [10] The goal of this systematic review and meta-analysis is to better define the toxicity profile of the anti-PD-1 monoclonal antibodies (pembrolizumab and nivolumab) in patients with solid tumors with particular attention to selected adverse events of interest.

## RESULTS

### Study inclusion and characteristics

**Figure 1:**
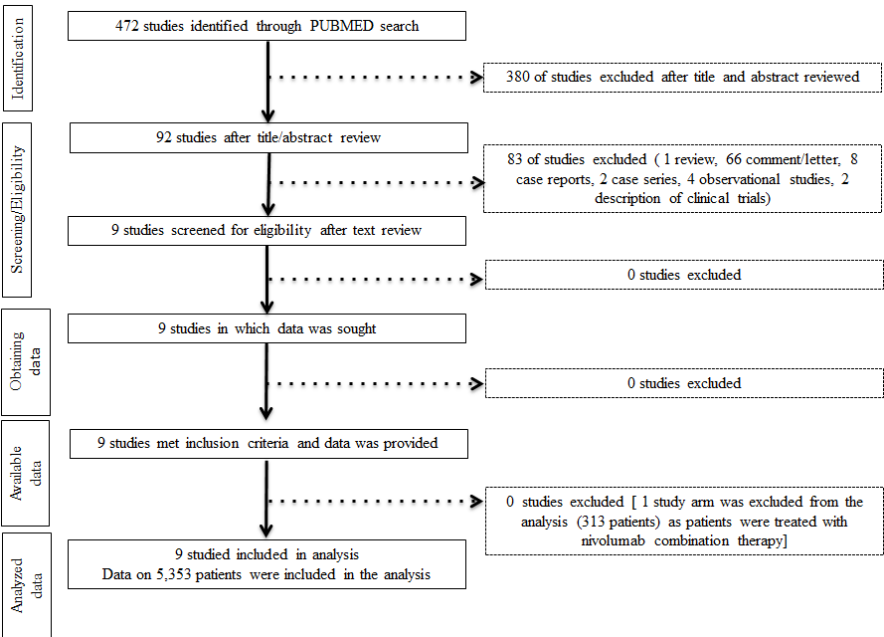
Study flow diagram

Initially our strategy yielded 472 publications through PubMed search. After screening of the study titles and abstracts 380 studies were excluded, as those were not prospective randomized trials. After text review 83 more studies were excluded for not meeting the inclusion criteria (Figure 1). Nine studies met the inclusion criteria and data were extracted. These studies comprised eight phase III and one phase II randomized trials. Six studies investigated nivolumab and three pembrolizumab. Five studies enrolled patients with metastastic melanoma, three NSCLC, and one RCC (Table 1).

**Table 1:** Characteristics of studies included in the analysis

First author (year)	Population (Line of therapy)	Number of patients evaluable for toxicity	Treatment ARM 1	Treatment ARM 2	Treatment ARM 3	Blinding	Pattern of randomization
Weber *et al.* (2015)[16]	Advanced melanoma (2^nd^ or 3^rd^ line)	370	Nivolumab 3mg/kg every 2 weeks	ICC Dacarbazine 1000mg/m2 every 3 weeks Or carboplatin AUC of 6 with paclitaxel 175mg/mg every 3 weeks	NA	Open label	Randomization ratio2:1, stratified by tumor PD-L1 status, *BRAF* status , and clinical benefit from previous. Permuted blocks (block size of six) within each stratum for randomization
Motzer *et al*. (2015)[3]	RCC (2^nd^ or 3^rd^ line)	803	Nivolumab 3mg/Kg every 2 weeks	Everolimus 10mg daily	NA	Open label	Randomization ratio 1:1, block size of 4, with stratification by region (United States or Canada, Western Europe, and the rest of the world), Memorial Sloan Kettering Cancer Center (MSKCC) prognostic risk group, and the number of previous antiangiogenic therapy regimens (one or two) for advanced renal cell carcinoma.
Borghaei *et al*. (2015)[12]	Non SCC NSCLC (2^nd^ line)	555	Nivolumab 3mg/Kg every 2 weeks	Docetaxel 75 mg/m2 every 3 weeks	NA	Open label	Randomization ratio 1:1, stratified by prior maintenance treatment (yes vs. no) and line of therapy (second line vs. third line)
Brahmer *et al*. (2015)[13]	SCC NSCLC (2^nd^ line)	260	Nivolumab 3mg/Kg every 2 weeks	Docetaxel 75 mg/m2 every 3 weeks	NA	Open label	Randomization ratio 1:1, stratified according to prior use of paclitaxel therapy (yes vs. no) and geographic region (United States or Canada vs. Europe vs. rest of the world [Argentina, Australia, Chile, Mexico, and Peru])
Robert *et al*. (2015)[14]	BRAF wild advanced melanoma (1^st^ line)	411	Nivolumab 3mg/KG every 2 weeks plus Dacarbazine-matched placebo every 3 weeks	Dacarbazine 1000mg/m2 every 3 weeks plus nivolumab matched placebo every 2 weeks	NA	Double blind, Placebo controlled	Randomization ratio 1:1, stratified by tumor PD-L1 status and metastasis stage (M0, M1a, or M1b vs. M1c, defined according to the tumor–node–metastasis system of the American Joint Committee on Cancer and the International Union against Cancer)
Larkin *et al*. (2015)[11]	Advanced melanoma (1^st^ line)	937*	Nivolumab 3mg/Kg every 2 weeks	Nivolumab 1mg/Kg every 3 weeks plus Ipilimumab 3mg/Kg every 3 weeks for 4 dose followed by Nivolumab 3mg/Kg every 2 weeks*	Ipilimumab 3mg/Kg every 3 weeks	Double blind, Placebo controlled	Randomization ratio 1:1:1, stratified by tumor PD-L1 status, *BRAF* mutation status, and American Joint Committee on Cancer metastasis stage (M0, M1a, or M1b vs. M1c)
Herbst *et al* (2015)[5]	NSCLC (2^nd^ line)	991	Pembrolizumab 2mg/Kg every 3 weeks	Pembrolizumab 10mg/Kg Every 3 weeks	Docetaxel 75mg/m2 Every 3 weeks	NO	Randomization ratio 1:1:1, stratified by ECOG PS (0 *vs* 1) and region (east Asia *vs* not east Asia), extent of PD-L1 expression. Treatment was allocated in blocks of six in each stratum
Ribas *et al* (2015)[15]	Ipilimumab resistant metastastic melanoma (2^nd^ line)	528	Pembrolizumab 2mg/Kg every 3 weeks	Pembrolizumab 10mg/Kg every 3 weeks	ICC (paclitaxel plus carboplatin, dacarbazine, or oral temozolomide)	Open label for chemo/ double blind for pembrolizumab dose	Randomization ratio 1:1:1, stratified by ECOG PS (0 *vs* 1), lactate dehydrogenase concentration, and *BRAF* mutation status. Block randomization with a block size of six in each stratum
Robert *et al 2*. (2015)[4]	Stage III or IV melanoma (1^st^ or 2^nd^ line only)	811	Pembrolizumab 10mg/Kg every 2 weeks	Pembrolizumab 10mg/Kg every 3 weeks	Ipilimumab 3mg/Kg every 3 weeks	Open label	Randomization ratio 1:1:1, stratified by ECOG performance status (0 versus 1), line of therapy (first versus second), and PD-L1 expression (positive versus negative).

Abbreviations: investigator's chemotherapy of choice (ICC), Non-small cell lung cancer (NSCLC), Squamous cell carcinoma NSCLC (SCNSCLC), Not applicable (NA)

* 313 patients were excluded from meta-analysis as there were treated with both nivolumab and ipilimumab

### Description of study participants

A total of 5,353 patients were evaluable for toxicity in all nine studies. Of those, 313 patients with advanced melanoma treated with the combination of ipilimumab and nivolumab were excluded from the analysis. [11] A total of 3205 patients with advanced stage solid tumors were randomized to anti-PD-1 therapy and 2148 patients were treated with standard non-anti-PD-1 therapy. Median age at study entry ranged from 59 to 64 years. Less than 1% of the patients accrued to the nine trials had ECOG performance status (PS) of 2 or higher. There were no significant imbalances with baseline patient characteristics between study arms within each trial.

### Study-to-study heterogeneity and publication bias

Inter-study heterogeneity I^2^ statistics were 92.2% for all grade AEs (*P* < 0.0001), 83.8% for grade 3/4 AEs (*P* < 0.0001), and 77.3% for all grade serious AEs (*P* = 0.004). There was no significant heterogeneity for the outcome of death. Egger's regression test was significant for all grade AEs (*P* = 0.014) indicating possible publication bias, but not for grade 3/4 AEs or all grade serious AEs. The Begg and Mazumdar test for publication bias was not significant for any of these outcomes.

### All grade, grade 3/4, and serious adverse events

**Figure 2:**
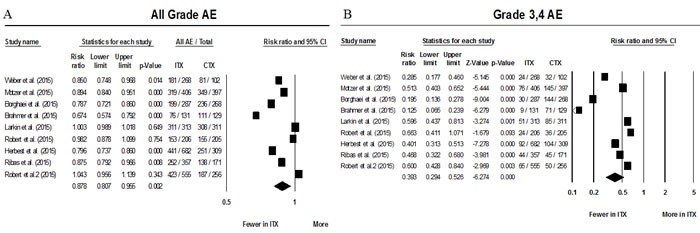
Forest plots of relative risks of all grade AEs (A) and grade 3&4 AEs (B) Abbreviations: Adverse events (AEs), immunotherapy therapy treatment arm (ITX), and control arm (CTX).

The number of all grade AEs and grade 3/4 AEs were available in all 9 studies. After accounting for inter-study heterogeneity meta-analysis showed a RR for all grade AEs of 0.87 (95% CI 0.81-0.95; *P* = 0.002) favoring treatment with anti-PD-1 antibodies. The absolute risk of grade 3/4 AEs was of 12.9% among patients treated with immunotherapy compared to 33.1% to standard of care approach (Figure 2).

Grade 3/4 AEs were also less frequent among patients treated with either immunotherapy when compared to standard of care with a RR of 0.39 (95% CI 0.29 - 0.53; *P* < 0.001) (Figure 2). Data on serious adverse events were available in only 4 of the 9 randomized studies. [12–15] RR for all grade serious AEs showed a trend favoring anti-PD-1 treatment but did not reach statistical significance (RR 0.56, 95%CI 0.31-1.04; *P* = 0.067).

All 9 nine studies reported the treatment-related death rates and in three of them there were no deaths reported. [14–16] The relative risk of death due to treatment related toxicity pooled from the remainder 6 studies was estimated at 0.45 (95% CI 0.19-1.09; *P* = 0.076) with a trending favoring less deaths among anti-PD-1 antibodies treated patients and absolute risk of death due to treatment related toxicity of 0.25% among these patients. In one study in which patients with advanced NSCLC were treated with pembrolizumab in the second line setting six of 11 deaths were documented among patients treated with pembrolizumab (causes of death: 3 pneumonitis and 2 pneumonia) (Table 2). On sensitivity analysis conducted by removing the two studies containing ipilimumab in the control arms (Larkin et al. and Robert et al.2) the correlations between PD-1 inhibitor and these endpoints (i.e.: all grade, grade 3/4, and serious adverse events) remained statistically unchanged. Finally all grade, and grade 3/4 toxicities were more common among patients treated with everolimus when compared to nivolumab (Motzer et al.).

**Table 2:** Treatment related deaths*

Publication	Population	ARM 1	ARM 2	ARM 3
Borghaei *et al*.(2015)[12]	Non SCC NSCLC / 2^nd^ line	Nivolumab 3mg/Kg every 2 weeks	Docetaxel 75 mg/m2 every 3 weeks	NA
Number of events	--	1 (encephalitis)	1 (febrile neutropenia)	NA
Brahmer *et al*.(2015)[13]	SCC NSCLC / 2^nd^ line	Nivolumab 3mg/Kg every 2 weeks	Docetaxel 75 mg/m2 every 3 weeks	NA
Number of events	--	0	3 deaths (one from interstitial lung disease, one from pulmonary hemorrhage, and one from sepsis)	NA
Motzer *et al*.(2015)[3]	RCC/ 2^nd^ or 3^rd^ line	Nivolumab 3mg/Kg every 2 weeks	Everolimus 10mg daily	NA
Number of events	--	0	2 (septic shock and 1 acute bowel obstruction)	NA
Larkin *et al*. (2015)[11]	Advanced melanoma/ 1^st^ line	Nivolumab 3mg/Kg every 2 weeks	Nivolumab 1mg/Kg every 3 weeks plus Ipilimumab 3mg/Kg every 3 weeks for 4 dose followed by Nivolumab 3mg/Kg every 2 weeks	Ipilimumab 3mg/Kg every 3 weeks
Number of events	--	1 (neutropenia)	0	1 (Cardiac arrest)
Herbst *et al* (2015)[5]	NSCLC	Pembrolizumab 2mg/Kg every 3 weeks	Pembrolizumab 10mg/Kg Every 3 weeks	Docetaxel 75mg/m2 Every 3 weeks
Number of events	--	3 (2 cases of pneumonitis and 1 of pneumonia)	3 (1 myocardial infarction, 1 pneumonia, 1 pneumonitis)	5 (1 case each of acute cardiac failure, dehydration, febrile neutropenia, interstitial lung disease, and respiratory tract infection)
Robert *et al 2*. (2015)[4]	Stage III or IV melanoma 1^st^ or 2^nd^ line only	Pembrolizumab 10mg/Kg every 2 weeks	Pembrolizumab 10mg/Kg every 3 weeks	Ipilimumab 3mg/Kg every 3 weeks
Number of events	--	0	0	1 (cardiac arrest secondary to metabolic imbalances associated with ipilimumab-induced diarrhea

Abbreviations: Abbreviations: Non-small cell lung cancer (NSCLC), Squamous cell carcinoma NSCLC (SCNSCLC), Not applicable (NA)

* Only the above mentioned 6 studies reported treatment related deaths

### Potentially irAEs

Of the 20 AEs of interest selected for RR pooled analysis only 14 were reported by at least 5 studies (Table 3). There was an absolute risk of thyroid disturbances of approximately 9% among patients treated with nivolumab or pembrolizumab. Patients treated with anti-PD-1 inhibitors had an increased risk of hyperthyroidism (RR 3.44; 95% CI 1.98-5.99; *P* < 0.001) and hypothyroidism (RR 6.79; 95% CI 3.10-14.84; *P* < 0.001) when compared to standard of care control arms. Five cases of adrenal insufficiency were reported among the patients treated with immunotherapy compared to none in the control arms.

**Table 3:** All grade AEs of interest reported by at least 5 studies

AE	Number of studies pooled	RR	95% CI	p	Absolute risk ITX	Absolute risk CTX
Creatinine elevation	7	2.83	1.17 - 6.85	0.02	1.06%	0.23%
Colitis*	8	1.06	0.33 - 3.44	0.92	1.09%	2.61%
Diarrhea	9	0.59	0.46 - 0.76	<0.001	11.61%	20.95%
Hyperglycemia	5	0.70	0.16 - 3.05	0.63	0.47%	2.19%
Hyperthyroidism	7	3.44	1.98 - 5.99	<0.001	3.03%	0.61%
Hypothyroidism	8	6.79	3.10 - 14.84	<0.001	6.52%	0.98%
Hypopituitarism	5	0.53	0.17 - 1.68	0.31	0.47%	1.28%
Infusion related reactions	6	0.54	0.27 - 1.09	0.09	0.84%	1.54%
Elevated AST and/or ALT	8	1.48	1.04 - 2.11	0.03	4.68%	2.98%
Mucosal inflammation/ stomatitis	5	0.17	0.10 - 0.30	<0.001	1.65%	13.78%
Pneumonitis	9	2.28	0.76 - 6.88	0.14	2.65%	3.31%
Pruritus	9	2.01	1.05 - 3.85	0.04	13.67%	11.64%
Rash	9	1.44	0.90 - 2.29	0.13	12.48%	12.43%
Vitiligo	5	4.92	2.07 - 11.69	<0.001	4.18%	0.88%

Abbreviations: Adverse events (AEs), relative risk (RR), confidence interval (CI), immune-therapy (ITX), control arm (CTX), alanine aminotransferase (ALT) aspartate aminotransferase (AST)

*Sensitivity analysis omitting Larkin et al and Robert et al 2 yielded a RR of 1.46 p =0.003 indicating higher risk of colitis in the ITX group

**Table 4:** All grade AEs of interest reported by less than 5 studies

AE	Publications	Number of cases ITX (Number of patients evaluable for toxicity)	Number of cases CTX (Number of patients evaluable for toxicity)
Nephritis	Ribas *et al*, Brahmer *et al* and Robert *et al* 2[4, 13, 15]	4 (1043)	1 (556)
Hepatitis or hepatocellular damage	Ribas *et al*, Herbst *et al*, Borghaei *et al* Robert *et a*l 2[4, 5, 12, 15]	16 (1881)	4 (1004)
Pancreatitis	Herbst *et al*[5]	3 (682)	0 (309)
Elevated lipase and or amylase	NR	NR	NR
Eye inflammation#	Ribas *et al* and Robert *et al* 2[4, 15]	6 (912)	0 (427)
Adrenal insufficiency	Herbst *et al*[5]	5 (682)	0 (309)
Neuropathy^&^	Borghaei *et al*, Brahmer *et al*, Herbst *et al*, Ribas *et al*[5, 12, 13, 15]	11 (1457)	82 (877)

Abbreviations: Adverse events (AEs), relative risk (RR), confidence interval (CI), immune-therapy (ITX), control arm (CTX), alanine aminotransferase (ALT) aspartate aminotransferase (AST). None of the studies reported elevated , Not reported (NR), lipase and or amylase elevations in the absence of pancreatitis

# Eye inflammation: uveitis, conjunctivitis or episcleritis

& Includes both sensory and motor

The absolute risk of colitis between the two groups was not statistically different with RR of 1.06; 95%CI 0.33-3.44; *P* = 0.92 (Table 3). Sensitivity analysis was performed excluding the 2 studies in which CTLA-4 inhibitor was utilized in the control arm for all irAEs. [4, 11] After removal of these studies the risk of colitis achieved statistical significance (RR 1.46; *P* = 0.03) indicating higher risk among PD-1 targeted treatment patients. Among the 16 cases of hepatitis or hepatocellular damage reported among patients treated with anti-PD-1 antibodies, at least 3 were considered of auto-immune etiology. Correlations between the remaining gastro-intestinal selected AEs and PD-1 inhibition remained unchanged.

All grade pruritus and vitiligo were more common in the pool of patients who received PD-1 inhibitors with RRs 2.10 and 4.92 respectively. Cases of vitiligo were reported in 5 of nine studies and only among patients with diagnosis of metastatic melanoma. Rash was present in approximately 12% of patients of both pooled groups. Furthermore there was no significant difference in the risk of all grade pneumonitis. Four cases of nephritis were reported among patients treated with anti-PD-1 therapy whereas only one was reported among patients treated in the control group. Eleven cases of neuropathy (motor either or sensory) were reported among patients treated with anti-PD-1 antibodies compared to 81 in the control groups.

### Time to onset and resolution of AEs in patients treated with anti-PD-1 antibodies

Robert et al. reported longer time to onset of grade 3 or higher AEs in the two pembrolizumab groups compared to the ipilimumab one (median time 59-64 *vs*. 39.5 weeks, respectively). [4] In the realm of selected AEs of possible immunological etiology, Brahmer et al. reported a median time to onset of grade 3 or higher gastrointestinal side effects with nivolumab of 91 weeks *versus* 1.1 weeks with docetaxel. [13] Similar results were observed by Borghaei et al. who reported median time to onset of grade 3 or higher gastrointestinal side effects with nivolumab of 11.7 weeks *versus* 1.1 weeks with docetaxel. [12] Time to onset of all grade hepatic toxicity was 1.9 *versus* 2.4 weeks in the docetaxel arm. [12]

The time to resolution of selected side effects was also reported by Borghaei et al. [12] Time to resolution of grade 3/4 gastrointestinal AEs was 2 *versus* 1week, pulmonary toxicity was 27.5 *versus* 9.1 weeks favoring nivolumab treatment as compared to docetaxel. In addition Robert et al. reported median time to resolution of nivolumab grade 3 and 4 induced endocrine toxicities 3.6 weeks, gastrointestinal 0.7 weeks, hepatic 13.3 weeks, and renal 6.1 weeks. [14] Larkin et al. reported median time to resolution of grade 3 and 4 skin, hepatic and pulmonary toxicities of 2.1, hepatic 7, pulmonary 2.3 weeks, respectively. [11] Of note, supportive treatment, which could have contributed to resolution of AEs was not described by the others (e.g., systemic steroid use) and could be a confounding factor for time to resolution of AEs.

Motzer et al. reported treatment-related AEs leading to permanent treatment discontinuation occurred in 31 of the 406 patients (8%) treated with nivolumab and in 52 of the 397 patients (13%) treated with everolimus among patients with Renal cell carcinoma. [3] Borghaei et al. and Brahmer et al. reported that discontinuation of nivolumab due to treatment-related AEs occurred less frequently with nivolumab than with docetaxel in 5% *vs*. 15% and in 3% *vs*. 10% of the patients with NSCLC respectively. [12, 13] Among patients with advanced melanoma PD-1 targeted antibody toxicities led to discontinuation in a low number of patients. Treatment-related AEs led to permanent discontinuation of these agents in 4.7-7.7% of patients whereas in the control groups treatment was discontinued in 11.7% (dacarbazine), 6% (chemotherapy) and 9.4-14.8% (ipilimumab). [4, 11, 14, 15]

## DISCUSSION

Prior published systematic reviews have addressed the prevalence of isolated organ specific toxicities of immune-check point inhibitors with different mechanisms of actions in their pooled analysis (i.e., anti-PD-1 and anti-CTLA-4 antibodies). [17, 18] In light of the growing clinical importance of PD-1 directed antibodies as exemplified by the publication of numerous randomized trials in 2015, we conducted a comprehensive systematic review and meta-analysis to refine the understand and assessment of the risk of AEs associated with approved PD-1 inhibitors (nivolumab and pembrolizumab) in patients with solid tumors. As a meta-analysis of large randomized clinical trials this study particularly improves the precision of prevalence estimates of potentially irAEs in these patients.

Nine randomized clinical trials met the inclusion criteria to enter our study and control arms included docetaxel, dacarbazine, everolimus, temozolamide, carboplatin combined with paclitaxel, and ipilimumab. These treatment regimens are considered standard of care options in each of the tumors studied (Table 1). A significant heterogeneity was observed between studies, which can be justified by three different histological tumor types, different disease phases among studies, and the diversity of treatment modalities in the controls arms. Treatment with pembrolizumab or nivolumab was overall better tolerated when compared to standard of care with significantly decreased risk of all grade AEs and grades 3/4. We also observed a trend favoring immunotherapy in regards to reduced AEs, serious adverse events and death due to treatment. The absolute risk of death from drug toxicity was of 0.25% for patients treated with anti-PD-1 agents, compared to 0.61% in the standard of care arms.

Early phase non-randomized clinical trials found higher absolute risks for some of these sided effects [e.g., pneumonitis (9-6%), hypothyroidism (7-12%), pancreatitis (15%), rash (12-22%), pruritus (23%)]. [19–25] Conversely, the results of our pooled analysis of the absolute and relative risks of potentially irAEs indicate that these AEs are less frequent than once thought. For instance, no case of pancreatitis was reported among the 3,205 patients treated with anti-PD-1 treatment and the absolute of risk pneumonitis was estimated at 2.65% in this cohort, which is much lower when compared to previous early phase studies. The vast majority of the potentially irAEs were either grade 1 or 2. Among the immunotherapy treated cohort there were only 31 cases (~1%) of grade 3/4 diarrhea (the most common selected AEs) and 17 cases of grade 3/4 colitis (~0.5%). It is important to highlight that the time to onset of grade 3 or higher AEs was longer with anti-PD-1 inhibitor compared to standard of care therapy (median 59-64 *vs* 39.5 weeks) respectively, which suggest the grade 3/4 toxicity occur with prolonged treatment exposure. [4]

Diarrhea was the most common potentially irAE with an absolute risk of 11.6% among patients treated with anti-PD-1 antibodies. However short the time to resolution of grade 3/4 gastrointestinal AEs (0.7-1 week) need to be weighed against the need for steroid based therapy in patients who present severe toxicities. [12, 14] Treatment related hypothyroidism and hyperthyroidism were also more common among patients treated with ant-PD-1 antibodies when compared to standard of care (RR 6.79 and 3.44% respectively). However the authors of the 9 clinical trials pooled did not provide detailed definition of this AE and cases of subclinical hypo or hyperthyroidism account for overestimation of clinically relevant thyroid disturbances. Taken together these results indicate that most patients are able to remain on anti-PD-1 directed therapy for prolonged periods of time without increased risk of clinically significant toxicities. This is particularly relevant for long-term responders who could potentially benefit from extended periods of treatment with PD-1 inhibitors. Nonetheless, despite the low frequency of potentially irAEs those can be serious events leading to hospitalization, invasive procedures (e.g., bronchoscopy for pneumonitis, colonoscopy for diarrhea/colitis, and even biopsy for nephritis) and death. Physicians should be cognizant of the particular AE profile of nivolumab and pembrolizumab, which may occur several weeks after the commencement of treatment and initiate supportive care accordingly. [6]

It is interesting to revisit that the degree of toxicity from EGFR and VEGF targeted drugs has been positively correlated with their efficacy among patients with solid tumors including colorectal, lung, renal cell carcinoma, and head and neck; so one could hypothesize a possible correlation between the presence of immune-related AEs and increased efficacy of PD-1 inhibitors. [26, 27] Thus far auto-immunity has been reported as favorable prognostic marker of outcome in patients with melanoma undergoing treatment with interferon alfa-2b and anti-CTLA-4. [28, 29] Nonetheless, Freeman-Keller et al. reported results of retrospective analysis of 148 patients with melanoma treated with nivolumab in which overall survival showed favorable correlation with presence of vitiligo (HR 0.18; 95%CI 0.036-0.94; *P* = 0.012). [30, 31] Further confirmation of auto-immunity correlation with improved outcomes among patients treated with PD-1 directed antibodies is awaited; and mechanistic insights on this association could shed light into future treatment development in solid tumors.

One of the limitations of the current study is that the data included do not represent individual participant data collection, which tempers our ability to perform exploration of additional correlations and interactions between anti-PD-1 immunotherapy and toxicities. It needs to be recognized that the strategy conducted during our literature search did not include Clinical trial registry database and meeting proceedings. However, the complete publication of randomized trials and full disclosure of AEs ARE not performed in these sources. Lastly, the lack of significant RR difference between the anti-PD-1 treated patients and the control groups could have been confounded by known overlapping toxicity profiles as there is well known risk of pneumonitis with paclitaxel (5-8%) and diarrhea (~30%) with everolimus treatment as well other standard of care treatments used such as ipilimumab. [7, 32–34] Furthermore, the obvious miscellany of tumor histological types could represent an additional confounding factor to our results. However the fact that the point estimates of relative and absolute risks seem to uniformly favor the toxicity profile of PD-1 inhibitors across different tumor types support the safety of these drugs for all endpoints analyzed except for thyroid and skin toxicities.

In summary, based on published randomized clinical trials our systematic review and meta-analysis indicate that pembrolizumab and nivolumab are associated with a relatively low risk of AEs. Hypothyroidism, hyperthyroidism, and pruritus are more frequent with anti-PD-1 monoclonal antibodies when compared to standard of care approaches. Vitiligo is also more common among patients with metastatic melanoma treated with these agents. As more toxicities are seen with more drug exposure these results suggest that more studies are warranted to define the true clinical benefit of prolonged treatment with anti-PD-1 inhibitors among the subset of patients with prolonged responses to therapy. There is low risk of diarrhea, nephritis, pneumonitis, hypophysitis and hepatitis with pembrolizumab and nivolumab therapy. Future drug development strategies combining nivolumab and pembrolizumab with other immunotherapy agents, targeted agents, and or chemotherapy will need to take into account the distinct toxicity profile of this class of agents.

## MATERIALS AND METHODS

### Search strategy

Randomized clinical trials (RCTs) were identified by a PubMed search using the following keywords or corresponding medical subject heading terms: “nivolumab” “pembrolizumab”. No filters or language limit was used to maximize search sensitivity. The database was searched for articles published until February 4^th^ 2016.

### Selection of trials and data extraction

Randomized trials were required to meet the following inclusion criteria: (i) at least one of the study arms consisting of nivolumab or pembrolizumab monotherapy; (ii) at least one of the control arms containing active therapy (without inclusion of anti-PD-1 inhibitor therapy).

For clinical trials meeting the inclusion criteria, the first author's name, date of publication, study phase, tumor type, type of anti-PD-1 inhibitor, pattern of randomization were collected for each study respecting guideline suggested by the Preferred Reporting Items for Systematic Review and Meta-Analyses of individual participant data: the PRISMA-IPD Statement. [35] The number of patients evaluable for toxicity and the number of adverse events (AEs) of study arms containing the same PD-1 inhibitor at different doses within the same clinical trial were summed for comparison with control arms (non-PD-1 containing regimen). According to the National Cancer Institute Common Terminology Criteria Adverse Events (NCI CTCAE) version 4.0, the number of all grade, grade 3 and 4, grade 5, and serious all grade and grade 3 and 4 toxicities were extracted. Serious adverse events were defined as death, life-threatening adverse drug experience, inpatient hospitalization or prolongation of existing hospitalization (for > 24 hours), a persistent or significant incapacity or substantial disruption of the ability to conduct normal life functions, a congenital anomaly/birth defect, an important medical event that may not result in death, based upon medical judgment, they may jeopardize the patient or subject and may require medical or surgical intervention to prevent one of the outcomes listed in this definition. Also, the number of all grade potentially irAEs were collected according to study arm [rash, pruritus, vitiligo, mucosal inflammation/stomatitis, diarrhea, colitis, hepatitis, elevations of alanine aminotransferase and or aspartate aminotransferase, hypopituitarism/hypophysitis, adrenal insufficiency, hypothyroidism, hyperthyroidism, hyperglycemia, non-infectious pneumonitis, eye inflammation (uveitis, conjunctivitis or episcleritis), increased creatinine level, nephritis, pancreatitis, elevation of lipase and or amylase, neuropathy and infusion related reactions (disorder characterized by adverse reaction to the infusion of pharmacological or biological substances infusion related reaction). In light of the expected low number of immune mediated adverse events all grades of selected AEs were extracted.

### Statistical methods

Data were analyzed using random effects meta-analysis for risk ratios. Heterogeneity across studies was analyzed using Q and I^2^ statistics. [36] Publication bias was analyzed using Begg and Mazumdar's tau test and Egger's regression intercept test. [37, 38]

### Sensitivity analysis

Pre-planned sensitivity analysis for all endpoints described above was conducted excluding any control arm containing non-PD-1 directed immunotherapy (i.e., CTLA-4 inhibitor ipilimumab) as these agents are likely to have overlapping toxicities with anti-PD-1 inhibitors.

Benedito A. Carneiro M.D., M.Sc.: In the past 2 years the author had research project funded, in whole or in part, by Bristol Myers Squibb.

Mark Agulnik M.D.: none.

Alfred W. Rademaker: none.

Sachin G. Pai M.D.: none.

Victoria A. Villaflor M.D.: In the past 2 years the author had research project funded, in whole or in part, by Celgene and Norvatis.

Massimo Cristofanilli M.D.: none.

Jeffrey A. Sosman M.D.: In the past 2 years the author received Honoraria from Novartis, Merck, Array, Genentech and had research project funded, in whole or in part, by BMV pharma.

Francis J. Giles M.D.: In the past 2 years the author received honoraria from Novartis and had research project funded, in whole or in part, by Bristol Myers Squibb, AbbVie and MedImmune.
